# Spatial evaluation of animal health care accessibility and veterinary shortage in France

**DOI:** 10.1038/s41598-022-15600-0

**Published:** 2022-07-29

**Authors:** Mehdi Berrada, Youba Ndiaye, Didier Raboisson, Guillaume Lhermie

**Affiliations:** 1 CIRAD, UMR ASTRE, Montpellier, France, ASTRE, CIRAD, INRAE, Univ Montpellier, Montpellier, Université de Toulouse, ENVT, Toulouse, France; 2 Department of Production Animal Health, Faculty of Veterinary Medicine, University of Calgary, Calgary, Canada

**Keywords:** Health policy, Health services

## Abstract

The decrease in the supply of veterinary healthcare in France adversely affects health of food-producing animals. In a One Health perspective, the health of people, animals and their shared environment are interconnected, and adequate supply of veterinary healthcare is required to ensure public health. Prevention of outbreaks and zoonotic diseases that may impact public health mobilizes a set of public policies, including strengthening veterinary workforce. These policies should be informed by quantification of animal health care accessibility, yet this has not been well characterized. The objective was to quantify the accessibility to veterinary healthcare for cattle, swine, and poultry sectors in France. A Two-Step Floating Catchment Area (2SFCA) approach was used to measure the level of accessibility to veterinary clinics. In the cattle sector, the 2SFCA score indicated relatively high accessibility in the north and south of France, but insufficient accessibility elsewhere. In the swine sector, there was high accessibility in the north east and in north of France, medium accessibility in the south west, and insufficient accessibility elsewhere. Finally, in the poultry sector, all regions had insufficient accessibility. Sensitivity analysis examining the effects of a change in spatial accessibility according to various travel time showed that the optimal threshold to compute 2SFCA score in cattle, swine and poultry sectors were respectively, 45, 60 and 60 min. According to a definition of “underserved area” derived from an official decree and the optimal thresholds to compute 2SFCA, the cattle, swine and poultry sector have on average 75.3, 89.9 and 98.3% underserved area, respectively. We provided evidence that the supply of animal health care was not sufficient and we proposed recommendations on how to assess animal health care accessibility, enabling modelling and visualization of the effects of potential public policies aimed at reducing veterinary shortages.

## Introduction

The veterinary profession is not exempt from shortages of personnel, particularly in rural areas, as observed in many other medical professions. In Europe, the Federation of Veterinarians of Europe (FVE) surveyed the shortage of veterinarians in rural areas in 2020^1^. Among 28 European countries surveyed, 78.5% are experiencing a shortage of veterinarians. However, this is neither exclusively a European concern, nor completely new.

In 2009, Andrus et al.^2^ revealed a pattern of significant future shortages in food animal veterinarians in the U.S. and Canada. The U.S. is facing a shortage of veterinarians, especially those with public health responsibilities, e.g., food animal, agricultural and public health practice^3^. Currently, there is a critical shortage of food animal veterinarians in both private and public practice, particularly in rural communities in the U.S. and insular areas^4^. Therefore, there is a need for more food animal and public health veterinarians, mainly in underserved rural areas^5^. A major difference in human and animal care systems is that in most developed countries, access to human health care is at least partly covered by mandatory public health insurance. These expenses are justified for reasons of public health and equity. However, in the veterinary sector, expenses are covered by animals’ owners, some of them holding private insurance to cover such expenses. Consequently, the animal healthcare market may act more as a perfect market, i.e., with supply matching demand, than the human health care market.

The economic definition of a shortage is a situation where the demand is greater than the supply at a market price. Applying this to the veterinary healthcare supply, a shortage consists of a lack of practitioners in a given area. The shortage may arise from two factors: first, this could be explained by a shrinking number of available veterinarians; second, this could be explained by increasing demand for healthcare. The veterinary profession has a unique feature; with one diploma, veterinarians are able to practice medicine and surgery on various species, spanning from companion animals (CA) to food producing animals (FPA). Consequently, practitioners may choose to specialize in a species, or to work in a so called mixed (CA and FPA) practice. Interestingly, this also enables them to change, during their career, the species of interest. Furthermore, many criteria may guide this shift, from economic to personal reasons.

Whereas geographic repartition of dogs and cats is relatively homogenous across French territory, the repartition of FPA is not. Rural areas obviously concentrate most of this animal population, with strong regional disparities with regards to species.

In Europe, the FVE reports that the shortage of veterinarians in rural areas is not caused by a lack of graduates, but is more linked to the preferences of veterinarians to work in urban locations^1^. Consequently, the problem of shortage of veterinarians affects mainly rural areas. Overall, these factors lead to a mismatch between veterinary supply and healthcare demand.

Consequences of veterinary shortages are three-fold. First, it may decrease farm profitability, due to a lack of veterinary supervision of the production process. Second, it poses animal welfare issues, as animals without adequate veterinary care may suffer or even die from diseases normally curable^6^. Third, it challenges the ability of the current veterinary healthcare system to manage endemic and epidemic animal diseases, with some of the most important being also zoonosis, e.g., Tuberculosis or Avian Influenza. Overall, an extensive veterinary shortage may affect the whole society, particularly if no alternative model of veterinary healthcare emerges.

This set of factors has led governments to consider measures to tackle veterinary shortages.

In the U.S., the National Veterinary Medical Service Act was adopted in 2003 to authorize the Secretary of Agriculture to conduct a loan repayment program regarding provision of veterinary services in shortage situations^7^. It is a way to nudge veterinarians to practice in rural areas. In addition, the Veterinary Public Health Workforce Expansion Act of 2005 was voted to award competitive grants to eligible entities to increase the number of veterinarians in the workforce reference. Furthermore, to promote recruitment of veterinarians, the U.S. Department of Agriculture has two programs: The Veterinary Medicine Loan Repayment Program and Veterinary Services Grant Program reference.

In 2013, the French Senate adopted a bill to address the veterinarian shortage in rural areas by promoting tutored internships. This measure aims to better integrate young veterinarians in rural areas. In December 2020, a law voted by the French government laid the juridical ground for subnational representations, e.g., regional assemblies and cities, to subsidize veterinarians willing to practice in underserved areas i.e., those with a veterinary shortage reference.

A preliminary step consists of defining eligible areas, and to do so, accessibility to animal health care should be quantified. However, there is currently no tool to measure spatial accessibility in animal health care. Accessibility consists of the distance or time between patient location and service points, whereas availability reflects the number of local service points from which a patient can choose. The combination between availability and accessibility is called spatial accessibility^8^.

Gravity models are commonly used to quantify spatial accessibility in urban and rural areas^9^. They are the most common formulation of the spatial interaction models^10^. The main benefit from gravity models is that it captures a large part of supply and demand match according to the accessibility and the availability. Other parameters are not included in gravity models as economic causes, personal values. The two-step floating catchment area (2SFCA) is a special and simplified case of the gravity-based method^8^.

The objective was to quantify spatial accessibility to veterinary healthcare for the cattle, swine and poultry sectors, the three major animal commodities in France, using the 2SFCA method.

## Material and methods

### Data

#### National inventory of animals (cattle, poultry and pig)

Two datasets were used to quantify demands for animal health care. All cattle farms were extracted from the French National Bovine Database Identification 2019 (BDNI), whereas all other swine and poultry farms were extracted from SIGAL (Sanitary Information System).

The BDNI database, managed by a specific office of the French Ministry of Agriculture and Food, was described^11^ and used^12^. All animals, farms, and farmers in the cattle sector are individually identified. The BDNI contains an identification number, date of birth, sex, farm of birth, geographic coordinates of farms, breed and date of first calving for females. These characteristics were used to compute Livestock Units (LU), which facilitates aggregation of livestock by taking into account the type of cattle (e.g., dairy or beef cows) and their age.

In addition, geographic coordinates and animal populations of all French swine and poultry farms in the SIGAL database 2019 were provided by the General Directorate for Food (DGAL) of the Ministry of Agriculture and Food Industry.

#### Veterinary population and workload

To quantify the supply of animal health care, we used a dataset from the 2019 Veterinarian National Order Database, which includes the number of veterinarians, the species for which they provide service, and the site where they practice. Each year, every practitioner is required to be registered in the National Order Database. We geocoded professional addresses to pinpoint geographic coordinates (longitude and latitude) of veterinarians.

Second, to estimate the percentage of full-time equivalent (FTE) dedicated to each sector for all veterinarians, we used data from a previous survey^13^. In brief, we distinguished veterinarians with a full-time equivalent in a sector ($FTE_{i, cattle}$ = 1 if veterinarian *i* works exclusively in cattle sector for instance) and veterinarians who work in various sectors $(FTE_{i, cattle} = x_{1} , FTE_{i,pig} = x_{2} , FTE_{i,poultry} = x_{3} \,where\,x_{1} , x_{2} , x_{3} \in ]0,1[\& \,x_{1} + x_{2} + x_{3} \le 1)$.

In this survey, 1457 veterinarians working in animal production were questioned regarding their hours of work, which constitutes a sample of 22.7% of the target population. Veterinarians reported the main animal species, the secondary animal species and potentially the tertiary animal species that they treated. From species declared by veterinarians in the National database, we established similarities between veterinarians to estimate FTE at the target population level.

### Computation of accessibility with the Two-step floating catchment area method

The 2SFCA index for each farm location was calculated with following 2 steps, depending on the sector.

In the first step, for each location of a veterinary clinic *j* in sector *s*, we computed a ratio $R_{j,s}$

1 $$R_{j,s} = \frac{{{\text{FTE }}_{j,s} }}{{\mathop \sum \nolimits_{{i \in \left\{ {d_{ij} \le d_{0} } \right\}}} D_{i,s} }}$$

where $R_{j,s}$ is the veterinary clinic of sector *s* to animal population of the same sector ratio at location *j* whose centroid falls within the catchment {$d_{ij} \le d_{0}$}, $FTE_{j,s}$ is the number of full-time equivalent vets in the clinic at location *j* in sector *s*, $D_{i,s}$ is the demand of farm at location *i* in the sector *s* within the catchment, and $d_{ij}$ is the travel distance between *i* and *j*.

In the second step, another catchment was centered on each farm of sector *s* at location *i*. Then, we computed the 2SFCA index by summing up $R_{j,s}$ ratios located in the floating catchment of that given farm location *i*.

2 $$A_{i,s} = \mathop \sum \limits_{{j \in \left\{ {d_{ij} \le d_{0} } \right\}{ }}} R_{j,s}$$

where $A_{i,s}$ is the spatial accessibility index at a given farm *i* of sector s to veterinary clinic of the same sector based on 2SFCA.

The spatial accessibility index $A_{i,s}$ depends on the sector, because the supply and the demand of cattle, swine and poultry sectors differ and should be considered on a case-by-case basis. ${\text{FTE }}_{j,s}$ corresponds to the supply and represents the work time of veterinarians in clinic *j* dedicated to sector *s*. $D_{i,s}$ corresponds to the demand of the farm *i* of the sector *s*. Differences among the three sectors in their demand for veterinary service is that in the cattle sector, demand is represented by Livestock Units (LU), whereas demand in the other two sectors is represented by the number of farms.

In our study, we assessed spatial accessibility at the “canton” level. Metropolitan France is divided into 96 departments which is a French administrative division where the largest in area is Gironde (10,000 km^2^) and the smallest is Paris (105 km^2^). The departments are subdivided into 1995 cantons and 34,836 communes. The canton is used as geographical unit in the present study since each vet clinic is often related to from one to several cantons, and we present results at both canton and department levels.

In our study, the service area was determined by time-distance (or travel time). To determine travel time, we used a network based on OpenStreetMap to measure the distance covered in a given travel time by car. An example of a 2SFCA calculation is available in Supplementary Fig. S1 and Supplementary Fig. S2.

Applying the 2SFCA method requires definition of the travel time threshold. Sensitivity analysis was conducted to examine the change in spatial accessibility according to various values of this parameter within reasonable ranges (15, 30, 45 and 60 min) in cattle, swine and poultry sectors. To compare between 2SFCA scores distributions generated by various travel time thresholds, we compared the minimum, 1st quartile, median, 3rd quartile, maximum, mean and variation of each distribution. Additionally, we compared the coefficient of variation (CV). In general, standard deviation is used to measure the variation of values in a single dataset; although this measures how far the average value lies from the mean, it does not allow a comparison of variation between two datasets. However, the coefficient of variation (CV), defined as the ratio of the standard deviation to the mean, is more commonly used to compare the degree of variation from one data series to another. The higher the CV, the greater is the level of dispersion around the mean. Expressed in percentage, the CV formula is: $CV = \frac{\sigma }{\mu }*100 \%$ where $\sigma$ is the standard deviation and $\mu$ the mean of a given distribution.

The spatial distribution of 2SFCA score in each sector was mapped. Then, spatial distributions generated by various travel time thresholds were plotted side-by-side to facilitate comparisons. To further analyze the difference between travel time thresholds, statistical results of all spatial distributions were summarized in a table.

In the cattle sector, we considered that a canton is underserved when < 2 FTE are available for every 10,000 LU. In the poultry and swine sectors, we considered a canton as underserved when < 2 FTE are available for every 200 farms.

## Results

### Cattle sector

**Figure 1:**
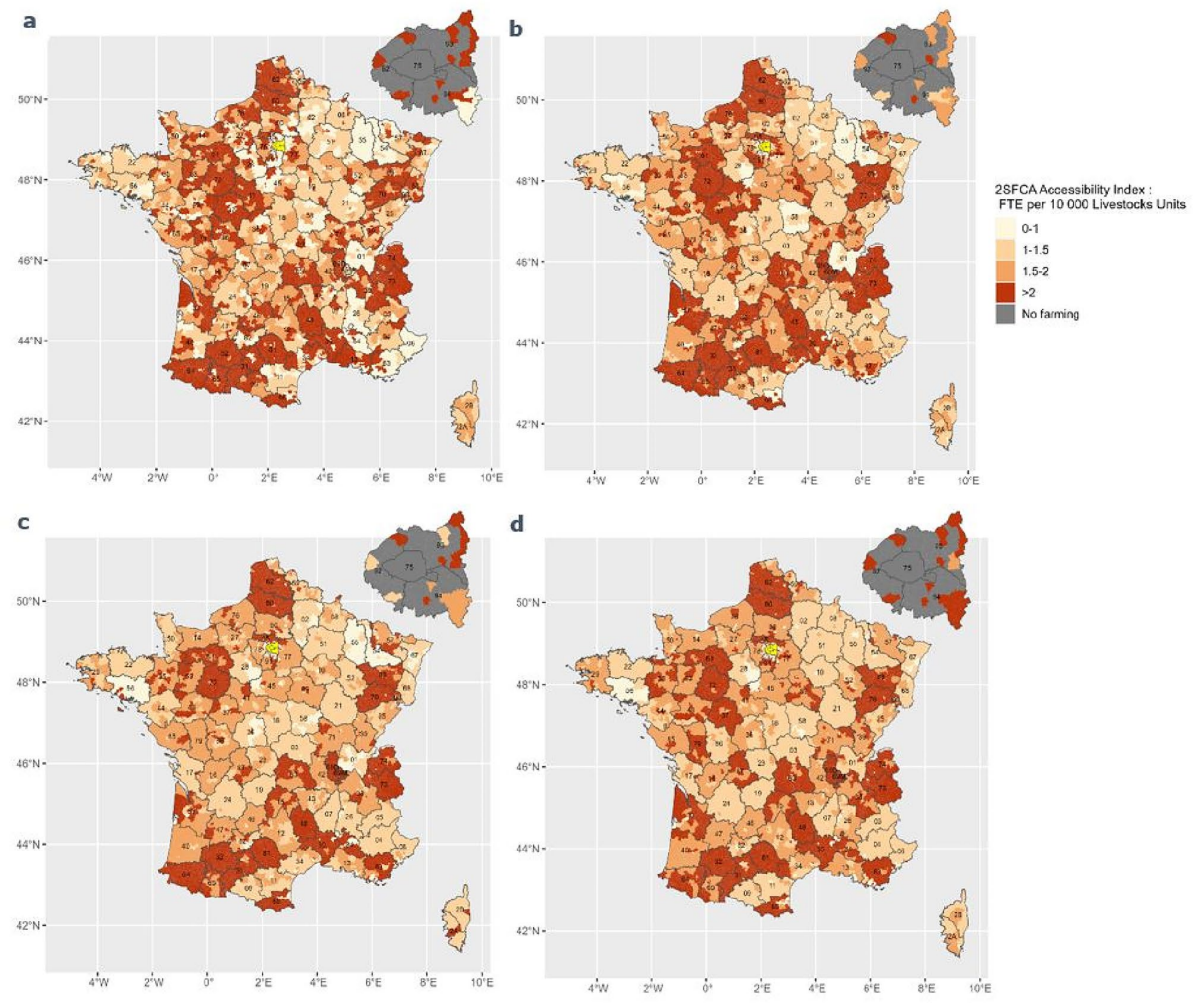
Spatial accessibility of the cattle sector to veterinarians in France for various travel time thresholds.

Supplementary Fig. S3 shows that in the spatial distribution of 174,364 cattle farms in France in 2019, there was relatively high density in the upper north west quadrant of the country—and at its center. Among 2035 veterinary clinics with cattle activity, 2056 FTE were dedicated to cattle with a high proportion in the north east and center of the country. Spatial accessibility maps calculated by 2SFCA according to various travel time thresholds in the cattle sector are compared (Fig. 1). Spatial accessibility categories were grouped, based on breaks that clearly identified animal health care needs of each canton. Dark orange areas indicated that supply in these regions was potentially able to satisfy the demand for animal health care (Fig. 1a–d), whereas white areas had critical shortages of supply to satisfy the demand (< 1 FTE per 10 000 LU).

Overall, the four travel time thresholds generated similar accessibility distribution patterns on a large scale, with relatively high accessibility in the north (departments 62, 80, 72, 61 and 76) and in the south (departments 64, 32, 31 and 81). There were 58, 59, 65, and 62% of cantons with a 2SFCA score < 2/10,000 (i.e., less than 2 FTE accessible and available per 10,000 LU) according to 2SFCA scores computed with 15, 30, 45, and 60 min thresholds. These cantons represent respectively 65, 67.4, 75.3 and 71% of surface area (Table 1). For further details, the percentage of underserved area by department in cattle sector is available in Supplementary Table S4.

**Table 1:** 2SFCA accessibility index distribution based on 4 travel time distances.

Travel time (min)	15	30	45	60
Minimum	0	0	0	0
First quartile	0.00008	0.00012	0.00012	0.00013
Median	0.00015	0.00016	0.00016	0.00016
Mean	0.00018	0.00018	0.00017	0.00019
3rd quartile	0.00023	0.00022	0.0002	0.00021
Maximum	0.00578	0.00294	0.0062	0.0421
Standard deviation	0.00025	0.00018	0.00018	0.00094
Coefficient of variation (%)	134	100	106	483
Area with < 2 FTE per 10,000 LU (%)	65.0	67.4	75.3	71.0

Differences in the distribution between the spatial accessibility index computed according to various travel time thresholds are detailed in Table 1. The accessibility scores of 2SFCA with a 15-min travel time threshold ranged from 0 to 0.00578 with an average of 0.00018 and standard deviation (SD) of 0.00025. The accessibility scores of 2SFCA with 30- and 45-min travel time thresholds had a narrower range from 0.00012 to 0.00294 and 0.0062, respectively, and the same SD of 0.00018. The accessibility scores of 2SFCA with a 60 min travel time threshold ranged from 0.00013 to 0.0421 with an average of 0.00019 and SD of 0.00094.

Based on the CV of the four scores, the spread of 2SFCA score was very different according to the travel time threshold. A larger threshold (from 15 to 45 min) reduced dispersion and consequently had a smaller CV; therefore, a larger threshold travel time generated stronger spatial smoothing. Spatial smoothing occurs when a concentric pattern and less variability are observed. However, with a 60-min travel time threshold, the CV started to rebound.

### Swine sector

The spatial distribution of 21,485 swine farms in France in 2019, represented in Supplementary Fig. S5 revealed relatively high density in the north east and in the extreme north west of the country (departments 57 and 67). Among the 182 veterinary clinics with swine activity, 138 FTE were dedicated to pigs, with a high proportion in the north east.

**Figure 2:**
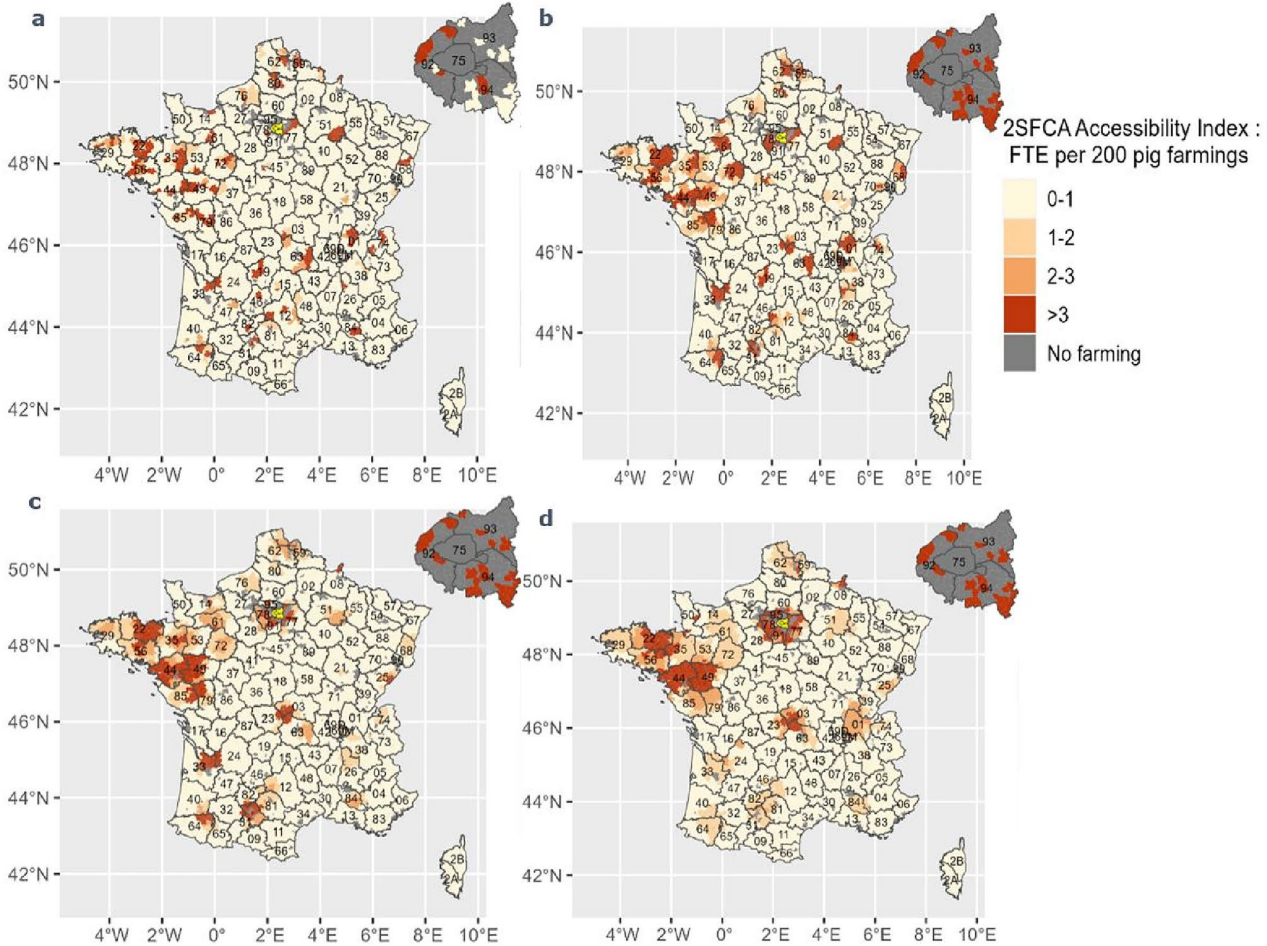
Spatial accessibility of the swine sector to veterinarians in France for various travel time thresholds.

The three travel time thresholds 30–60 min in Fig. 2b–d generated similar accessibility distribution patterns with relatively high accessibility in the north east (departments 22, 56, 35, 44, 49, 85) and in the north (departments 62 and 59) and medium accessibility in the south west. Figure 2a represents the 2SFCA score with a 15 min threshold; it had a different pattern compared to the other thresholds. Irrespective of the travel time threshold, there was a high proportion of underserved cantons, with 71, 66, 66, and 68% of underserved cantons with a 2SFCA score < 2/200(less than 2 FTE accessible and available per 200 farms) according to 2SFCA scores computed with 15, 30, 45, and 60 min thresholds. These cantons represent respectively 93.1, 90, 90.1 and 89.9% of surface area (Table 2). For further details, the percentage of underserved area in swine sector by department is available in Supplementary Table S6.

**Table 2:** 2SFCA accessibility index distribution based on 4 travel time distances.

Travel time (min)	15	30	45	60
Minimum	0	0	0	0
First quartile	0	0	0	0
Median	0	0	0.00053	0.0022
Mean	0.0065	0.0053	0.005	0.0052
3rd quartile	0	0.0037	0.0055	0.0064
Maximum	0.18	0.063	0.043	0.04
Standard deviation	0.027	0.013	0.0092	0.0083
Coefficient variation (%)	405	237	187	159
Area with < 2 FTE per 200 exploitations (%)	93.1	90.0	90.1	89.9

Differences in the distribution between the spatial accessibility index computed according to various travel times in the swine sector are shown (Table 2). The accessibility scores of 2SFCA with a 15 min travel time threshold ranged from 0 to 0.18 with an average of 0.0065 and SD of 0.027. Accessibility scores of 2SFCA with 30, 45 and 60 min travel time thresholds had a narrower range from 0 to 0.063, 0.043 and 0.04 respectively, with SD of 0.013, 0.0092 and 0.0083.

The CV of the four scores indicated decreased variability according to the length of the travel time threshold. Similar to the cattle sector, a larger threshold travel time generated stronger spatial smoothing. The CVs of 2SFCA score for 15, 30 and 45 min thresholds were higher compared to CVs in the cattle sector.

### Poultry sector

Supplementary Fig. S7 shows the spatial distribution of 28,136 poultry farming in France in 2019 reveals a relatively high density in the north east and in the south east. Among the 189 veterinary clinics with poultry activity, 134 FTE were dedicated to poultry.

**Figure 3:**
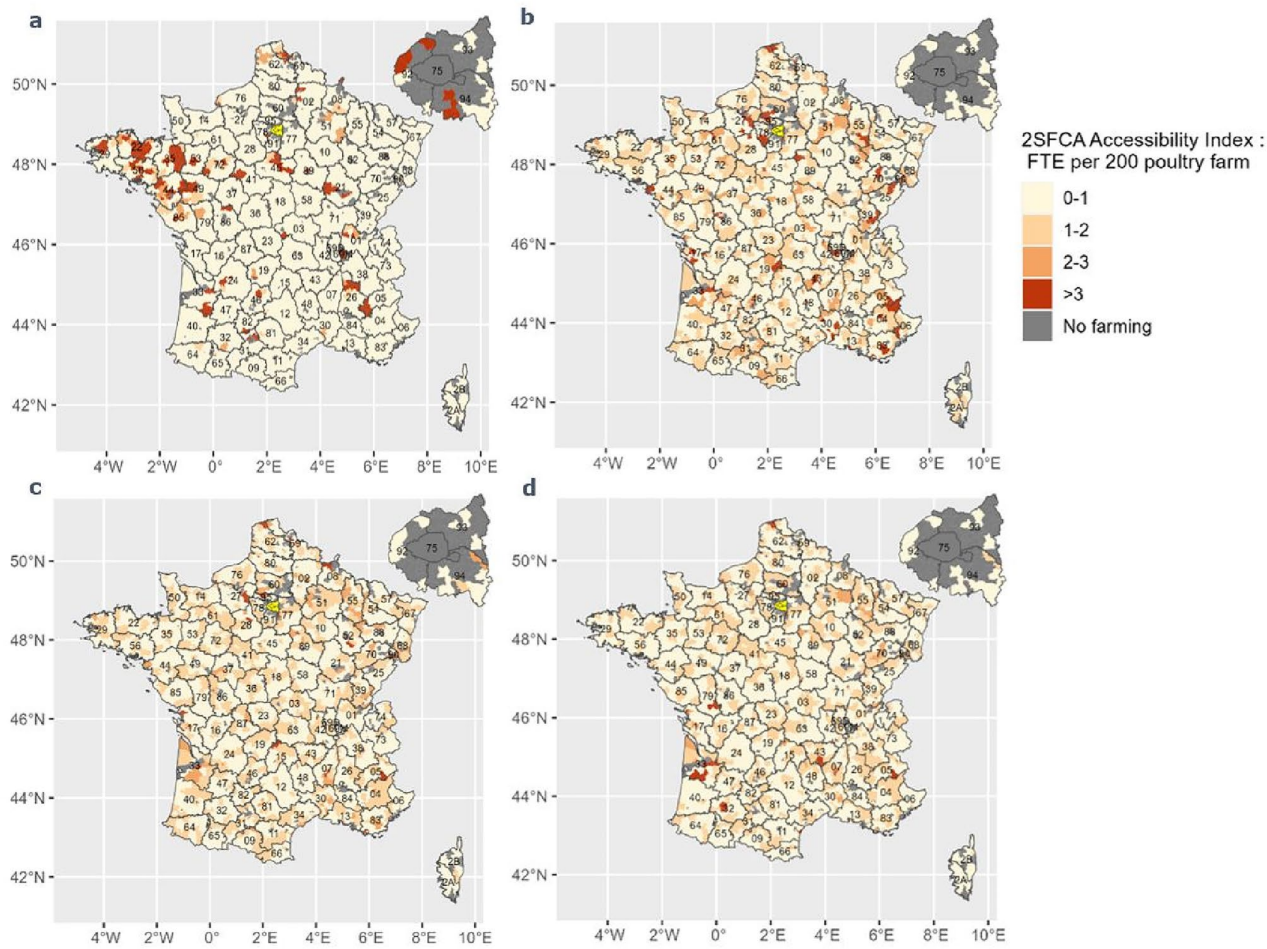
Spatial accessibility of the poultry sector to veterinarians in France for various travel time thresholds.

The three travel time thresholds 30–60 min in Fig. 3b–d highlight similar accessibility distribution patterns. Figure 3a represents the 2SFCA score with a 15 min threshold; it had a different pattern compared to the other thresholds. There were 71, 72, 74 and 75%, respectively of underserved cantons with a score < 2/200 (less than 2 FTE accessible and available per 200 farms) computed with 15, 30, 45 and 60 min thresholds. These cantons represent respectively 93.7, 92.9, 97.2 and 98.3% of surface area (Table 3). For further details, the percentage of underserved area by department in poultry sector is available in Supplementary Table S8.

**Table 3:** 2SFCA accessibility index distribution based on 4 travel time distances.

Travel time (min)	15	30	45	60
Minimum	0	0	0	0
First quartile	0	0.00166	0.00229	0.00265
Median	0	0.00350	0.00391	0.00406
Mean	0.00531	0.00451	0.0044	0.00431
Third quartile	0	0.00589	0.00563	0.00542
Maximum	0.17963	0.02649	0.02226	0.01608
Standard deviation	0.0235	0.00455	0.00336	0.00267
Coefficient variation (%)	442	100	76	61
Area with < 2 FTE per 200 exploitations (%)	93.7	92.9	97.2	98.3

Differences in the distribution between the spatial accessibility index computed according to the various travel times in the swine sector are shown (Table 3). Accessibility scores of 2SFCA with a 15 min travel time threshold ranged from 0 to 0.17 with an average of 0.0053 and standard deviation of 0.023. The accessibility scores of 2SFCA with a 30 min travel time threshold ranged from 0.0016 to 0.02 with an average of 0.0045 and SD of 0.0045. The accessibility scores of 2SFCA with a 45 min travel time threshold ranges from 0.0023 to 0.022 with an average of 0.0044 and SD of 0.0034. The accessibility scores of 2SFCA with a 60 min travel time threshold ranged from 0.0026 to 0.016 with an average of 0.0043 and SD of 0.0027.

As with cattle and swine sectors, a larger threshold travel time generates stronger spatial smoothing. Similarly, the CV in the poultry sector was relatively high, because many cantons have a null accessibility score, thereby increasing variation.

## Discussion

This study analysed spatial accessibility of veterinarians in cattle, swine and poultry sectors with 2SFCA method. To measure the accessibility index based on 2SFCA, a distance measurement for the floating catchments must be defined. Floating catchments are areas drawn around a location and could be defined by several distances, including Euclidean distance^14–16^, Manhattan distance, network distance and travel time-distance^17,18^. However, the most realistic and appropriate measure is time-distance^19^, the travel time required to go from one location to another. Four reasonable ranges (15, 30, 45 and 60 min) were defined, based on prior studies. As Wang noted^20^, the travel time threshold should be selected according to the real world spatial behavior. In this respect, a survey was conducted by Veterinarian National Order^13^ which showed that veterinarians travel on average 40 min by car in the cattle sector, whereas travels in the swine and poultry sectors are generally longer travels. Therefore, the four ranges (15, 30, 45 and 60 min) were retained. Interestingly, acceptable travel time thresholds can vary, depending on the type of services as well as the condition of the transport infrastructure^21^. For example, 40 min to reach dental and psychiatric care in the USA was considered appropriate, whereas for primary care, the suggested threshold was 30 min^22^. Several studies considered the threshold of 30 min in developed countries as appropriate^8,15,21^.

The sensitivity analysis was conducted to provide a global vision of the spatial accessibility distribution. This is important because measures of spatial accessibility may be biased if they are based on only one travel time threshold, due to variations among sectors in their needs. In the cattle sector, there was a relatively high accessibility in both the north (departments 62, 80, 72, 61 and 76) and in the south (departments 64, 32, 31 and 81). The sensitivity analysis of travel time thresholds had similar distribution patterns at a large scale, with high accessibility in the north and in the south west. However, different levels of dispersion were observed among the four 2SFCA scores, based on the CV. The increase in travel time threshold from 15 to 45 results in more veterinarians being available, with fewer non-null accessibility scores and spatial smoothing, which means less variation. In contrast, with 60 min travel time threshold, some isolated cantons that have a null accessibility score with 15–45 time thresholds become in a catchment with a large number of veterinarians. What explains that the CV started to rebound is the size of farms in these isolated cantons. Since the accessibility score is based on a ratio supply/demand, cantons with a very small demand has an accessibility score that goes sharply from 0 (no veterinarian accessible in 15–45 time thresholds) to a large score when the time threshold becomes too large and covers some veterinarians. It is the case for example in some cantons in the department 92 where the Livestocks Units are very low, but with a 60-min travel time threshold, the accessibility score becomes very high and leads to an increase in the CV. Therefore, the 60-min threshold should not be retained to avoid these outlier cantons. Consequently, 45 min was regarded as the optimal travel time threshold according to the survey conducted^13^ for this purpose. Overall, regardless of the travel time threshold, the percentage of underserved area is very high, from 65 to 75.3%. To pinpoint the most underserved department, the supplementary Table S4 will be helpful for policy makers.

In the pig sector, a high accessibility in the north east (departments 22, 56, 35, 44, 49, 85) and in the north (departments 62 and 59) and medium accessibility in the south west. Furthermore, the pattern of 2SFCA score computed with 15 min travel time threshold differed from other travel time thresholds, as 15 min in this sector was not adapted to veterinarians’ travels. In that regard, the most suitable travel threshold was 45 or 60 min, the average travel time for veterinarians in this sector. Furthermore, the sensitivity analysis in the swine sectors highlighted greater variability of 2SFCA scores for 15, 30 and 45 min thresholds compared to the cattle sector. This was attributed to the pig sector having many cantons with null accessibility score (the 2SFCA score for 15 and 30 min thresholds have respectively 83% and 63% of null values in pig sector) since the veterinary clinics that practice in the swine sector are not as widely distributed as those in the cattle sector. In this sector, 60 min as travel time threshold is recommended. Unlike the cattle sector, there was no outlier cantons with a small demand and significant supply with 60-min travel time threshold. The percentage of underserved area is critical, from 89.9 to 93.1% according to the different travel time thresholds.

In the poultry sector, there was no clear, general pattern of 2SFCA score. Along with the swine sector, the most suitable travel threshold was 45 or 60 min. As well as the cattle and swine sectors, the sensitivity analysis in the swine sector raised a higher variation of 2SFCA scores as travel thresholds increased, due to spatial smoothing. Many cantons had null accessibility scores, due to the lack of veterinarians in this sector. Regarding the veterinarians’ travels in this sector and in view of the 2SFCA score variation, 60 min as travel time threshold was the most appropriate. The percentage of underserved area is also critical, from 92.9 to 98.3% according to the different travel time thresholds.

In an official decree^23^, the French Ministry of Agriculture and Food defined acceptable veterinary coverage for common veterinary procedures, prescribing and drug delivery purposes, when more than 2 FTE are available for every 10,000 LU in the cattle sector. In the poultry and swine sectors, an acceptable veterinary coverage consists of > 2 FTE available for every 200 farms. We used these thresholds to interpret underserved areas.

Access to healthcare could be defined as the opportunity to use health care. A clear way of thinking about access is to consider access in terms of dimensions. These dimensions are barriers that provide patient to have access to health care^9^. Therefore, to assure the access to health care, policy makers require reliable measures to tackle these barriers. In human healthcare, there are five barriers: availability, accessibility, affordability, acceptability and accommodation^24^. The first two barriers are spatial, whereas the last three are a-spatial. Availability reflects the number of local service points from which a patient can choose. Accessibility is the distance or time between patient location and service points. Affordability reflects the economic capacity for people to spend resources and time to use appropriate services^25^. Acceptability is the professional values, culture and gender. Accommodation concerns the hours of opening, the appointments and all the mechanisms to use services. The combination between availability and accessibility is called spatial accessibility^8^ and is focused in the present work. The measure of spatial accessibility can rely on 4 items: provider-population ratios, distance to nearest provider, average distance to set of providers and gravity models.

Gravity models is widely used because they provide the most valid measures of spatial accessibility in urban and rural areas^9^. First, gravity models are better than provider to population ratio method because gravity models solve the borders effects issue. Second, gravity models consider the number of physicians in the study area contrary to distance to nearest supply. Third, comparing to the average travel distance method, gravity models do not overweight providers located in the periphery of the study area. Three limitations could be addressed to Gravity models. First it does not consider the interactions outside the catchments. Second, the threshold distance needed to calculate the catchments makes this indicator complicated to calculate. Third, the gravity model tends to give high accessibility scores in poor-access areas^8^.

According to Luo and Wang^8^, the major difference between gravity models and 2SFCA is that the former defines the accessibility as a continuous measure whereas the latter uses a dichotomous measure. Regardless, the main reason why 2SFCA is preferred among gravity models is that it involves less computation and programming and is more intuitive. The 2SFCA method combines availability and accessibility and overcomes the borders effects. Moreover, the 2SFCA approach considers interaction between demand and supply based on time-distance. Generally, the centroid of the catchment areas in the 2SFCA method is the center of some city or county. The particularity of our study is that we computed spatial accessibility from each farm. Then, we computed the spatial accessibility indexes average in each canton. Indeed, computing indexes with each farm in a county as the center before computing the average is more accurate than taking only the center of a county. Nevertheless, 2SFCA is a dichotomous measure that considers all the supply in a catchment area as homogeneous and does not account for distance decay within a catchment as gravity models.

However, this study had some limitations. Firstly, we quantified the demand of animal health care by the number of LU in cattle sector and the number of farms in the swine and poultry sectors. Further studies are needed to evaluate the “true” demand, as the number of veterinary interventions instead of LU or the number of farms, and differentiate between the size of farms in pig and poultry sectors. Yet, veterinary clinics keep these data confidential.

Secondly, since 2SFCA is a dichotomous measure, an alternative way to measure the spatial accessibility would be using the E2SFCA (Enhanced Two-Step Floating Catchment Area) where catchments are broken into discrete zones (e.g., 0–10, 10–20, and 20–30 min) with constant weightings applied in each zone^17^. Our data based on isochrones allow us only to know if a farm is at 15, 30, 45 or 60 min from a veterinary clinic. Yet, E2SFCA approach requires the exact distance computation between each farm and each veterinary clinic. To alleviate this shortcoming, we carried out a sensitivity analysis in order to identify clearly the difference between 2SFCA score in various travel time thresholds.

Finally, the results of this study should be interpreted with caution because the 2SFCA method is generally used to analyze spatial accessibility to human health care. Hence, applying 2SFCA method in animal health care has several consequences. First, the veterinarians are those who travel to livestock while generally the patient are those who go to the care resources. Regardless, the 2SFCA philosophy remains relevant. Indeed, numerous studies have used 2SFCA in human health to measure accessibility to urban fire service^26^, or to emergency medical services where patients do not travel. Second, an enhanced method of measuring accessibility to human health care was proposed by integrating multiple transportation modes, especially in urban areas. In the animal health care accessibility, veterinarians always travel by car. Hence, we computed the travel distance only by car. Third, we did not quantify the animal health care supply as the number of veterinarians but by full-time equivalent. The purpose is to avoid overestimating veterinarian availability. We separated between animal sectors because veterinarians perform activities in specific sectors even if all French veterinarians are sufficiently qualified to treat all species. We exploited a survey that estimates for each veterinarian the percentage of FTE dedicated to each sector.

Despite the obvious differences between the accessibility to animal health care and human health care, 2SFCA remains relevant if we pay close attention to quantify demand and the supply according to the sector and to select a suitable travel time threshold which depends on the needs of the sector.

The method used in this study provides interesting support to identify the most underserved cantons and to improve equity in access to animal health care. We conducted this study at a canton level, but since departments are likely the administration unit where public policies can be decided, we drew departments’ boundaries and printed the department number on the maps. The supplementary tables S4, S6 and S8 should help policy makers at the head of a department to take appropriate measures regarding the situation of their cantons. The present study supported the Department of Agriculture and Food to determine eligible departments to get financial supports. Department of Agriculture and Food defined eligible departments in cattle sector as the department with more than 3% of cantons’ surfaces with a 2SFCA index less than 2/10,000 (i.e., less than 2 FTE accessible and available for 10,000 LU). However, in the poultry and pig swine sectors, departments are eligible when more than 3% of cantons’ surfaces have a 2SFCA index less than 2/200 (i.e., less than 2 FTE accessible and available for 200 farms).

## Conclusions

This study quantified the spatial accessibility to animal health care in cattle, swine and poultry sectors in France using the 2SFCA method. It provides policy makers an overview of accessibility to animal health care at various geographic levels and identifies underserved areas. Each policy maker can decide a cut off to define areas with low-, medium- or high-accessibility. Eligible regions to financial supports were determined by this method. Indeed, it was agreed in an official decree that underserved cantons in cattle sector are those having less than 2 FTE per 10,000 livestock units and underserved cantons in pig and poultry sectors are those having less than 2 FTE per 200 farms. According to this decree, the cattle, swine and poultry sector have on average 75.3, 89.9 and 98.3% underserved area with 45, 60 and 60 min travel time thresholds, respectively. Additionally, this study used a flexible method for quantifying the spatial accessibility to animal health care in the cattle, swine and poultry sectors; this could be adapted to other animal production sectors. It should be noted that this method could be conveniently applied to other countries by choosing appropriate geographic breakdowns.

## Supplementary Information

Supplementary Information.

## Data Availability

The data that support the findings of this study are provided by the General directorate for food (DGAL) of Ministry of Agriculture. Restrictions apply to the availability of these data, and so are not publicly available.
