# Supplementary material for: Spatial evaluation of animal health care accessibility and veterinary shortage in France
Source: Sci Rep. 2022 Jul 29;12:13022. doi: 10.1038/s41598-022-15600-0 (PMC9338267; doi:10.1038/s41598-022-15600-0)
Supplement: Supplementary file 1 — Supplementary Information. [file 41598_2022_15600_MOESM1_ESM.docx]

# Supplementary Materials

**Spatial Evaluation of Animal Health Care Accessibility and Veterinary Shortage in France**

**Mehdi Berrada**^1,*^, **Youba Ndiaye**^1^, **Didier Raboisson**^1^ , **and** **Guillaume Lhermie**^1,2^

^1^CIRAD, UMR ASTRE, Montpellier, France, ASTRE, CIRAD, INRAE, Univ Montpellier, Montpellier, Université de Toulouse, ENVT, Toulouse, France

^2^Department of Production Animal Health, Faculty of Veterinary Medicine, University of Calgary, Canada

^*^[mehdi.berrada@envt.fr](mailto:mehdi.berrada@envt.fr)

**Example of 2SFCA calculation**

An example of catchment areas computed with R software is represented in Supplementary Fig. S1. Yellow and red catchment areas represent the catchment area of both veterinary clinics AHC1 and AHC2. The blue circles correspond to the livestock where the size of the circle reflects the number of LU. Note that livestock at the intersection of the two catchments are served by both AHC1 and AHC2.

**Supplementary Figure S1: Cacthment areas of two Animal Health Clinics in the first step of 2SFCA calculation.**


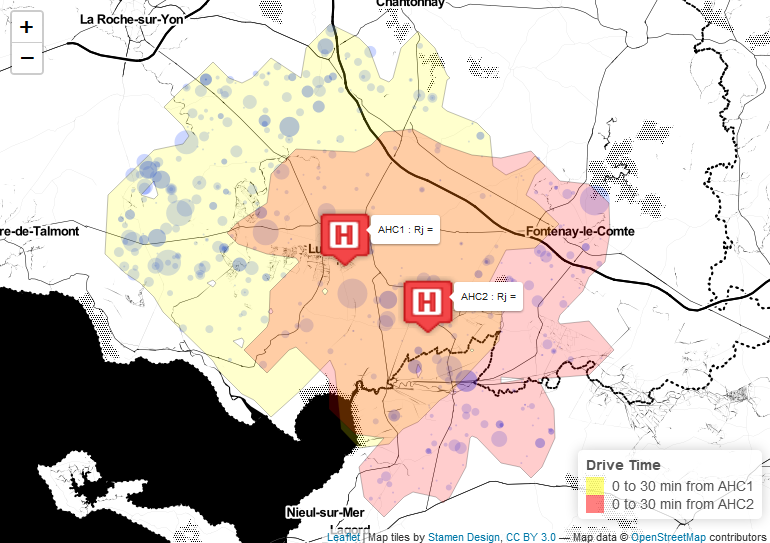


This figure was created by R software[14] version 4.0.3.

For each veterinary clinic we computed the Rj ratio as it has been shown in the first step of the 2SFCA.

In the second step of 2SFCA, the accessibility of each livestock is calculated and is represented in Supplementary Fig. S2. In this step we summed all the Rj ratios within the catchment area that have livestock as centroid. In the following figure we depicted only three catchment areas around three livestock to simplify.

**Supplementary Figure S2: Cacthment areas of three livestocks in the second step of 2SFCA calculation.**


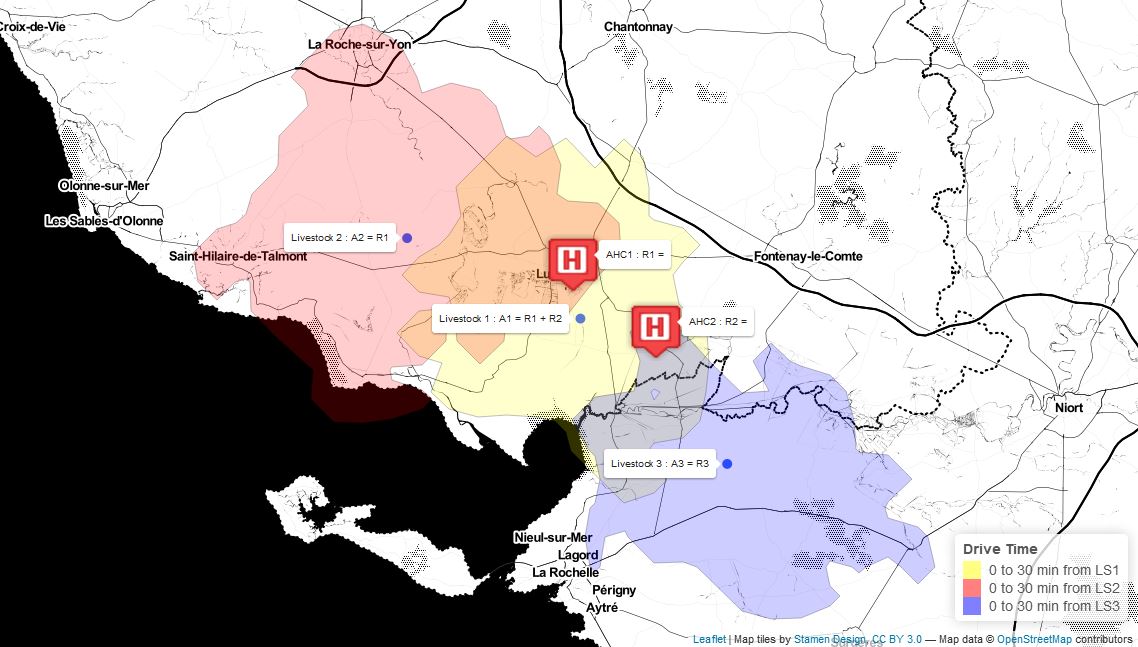


This figure was created by R software[14] version 4.0.3.

The 2SFCA spatial accessibility index Ai depends on the number of AHC around. Since the Livestock LS2 et and LS3 have only one AHC in their respective catchment, the index Ai corresponds to the only ratio in each catchment. Regarding the livestock LS1, the 2SFCA accessibility index corresponds to the sum of both R1 and R2 since both AHC are within the catchment area around the LS1.

**Descriptive statistics**

**cattle sector**

**Supplementary Figure S3: Distribution of exploitations and Full-Time Equivalent in cattle sector**


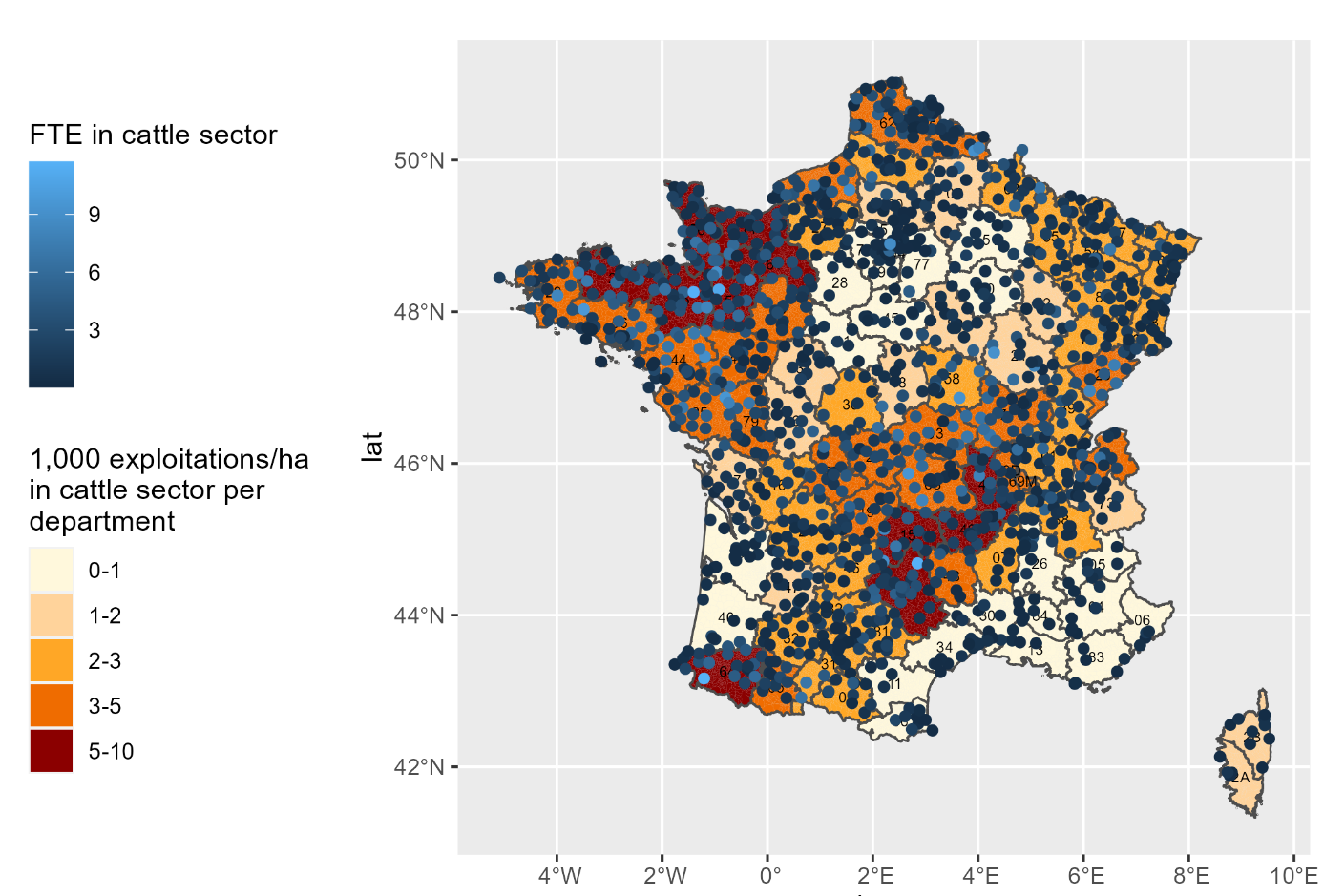


This figure was created by R software[14] version 4.0.3.

**Supplementary Table S4 : Percentage of underserved cantons by departement in cattle sector according to travel time thresholds.**

| departement | underserved cantons 15 min threhsold (%) | underserved cantons 30 min threhsold (%) | Underserved cantons 45 min threhsold (%) | Underserved cantons 60 min threhsold (%) |
| --- | --- | --- | --- | --- |
| 1 | 100 | 100 | 100 | 85.9 |
| 2 | 97.4 | 100 | 100 | 100 |
| 3 | 90.7 | 100 | 100 | 97.2 |
| 4 | 77.3 | 100 | 100 | 100 |
| 5 | 100 | 100 | 100 | 100 |
| 6 | 100 | 100 | 100 | 100 |
| 7 | 84.1 | 100 | 100 | 100 |
| 8 | 93.8 | 100 | 97.9 | 100 |
| 9 | 44.7 | 91.6 | 100 | 100 |
| 10 | 100 | 98.2 | 100 | 100 |
| 11 | 100 | 100 | 100 | 100 |
| 12 | 87.4 | 92 | 97.3 | 96.7 |
| 13 | 11.9 | 77.6 | 100 | 100 |
| 14 | 69.6 | 82 | 96.3 | 97.1 |
| 15 | 71.1 | 54.4 | 44 | 36 |
| 16 | 60.5 | 98.1 | 98.1 | 93.5 |
| 17 | 98.8 | 100 | 100 | 100 |
| 18 | 100 | 100 | 100 | 100 |
| 19 | 97.3 | 95.6 | 100 | 100 |
| 21 | 100 | 100 | 100 | 100 |
| 22 | 100 | 100 | 100 | 100 |
| 23 | 93.1 | 100 | 100 | 100 |
| 24 | 93 | 100 | 100 | 100 |
| 25 | 94.7 | 100 | 100 | 100 |
| 26 | 99.2 | 100 | 100 | 100 |
| 27 | 70.7 | 93.8 | 96.7 | 96.9 |
| 28 | 82 | 100 | 100 | 100 |
| 29 | 98.6 | 95.9 | 97.1 | 97.1 |
| 2A | 100 | 100 | 80.4 | 100 |
| 2B | 99.6 | 100 | 95 | 100 |
| 30 | 4.9 | 2.9 | 2.9 | 0 |
| 31 | 3.5 | 1.5 | 1.9 | 3.5 |
| 32 | 1.7 | 0 | 0 | 0 |
| 33 | 26.6 | 8.3 | 60.6 | 22.7 |
| 34 | 36.3 | 30.5 | 100 | 75.8 |
| 35 | 83.1 | 74.3 | 73.7 | 39 |
| 36 | 81.3 | 85.2 | 98.7 | 98.7 |
| 37 | 11.8 | 11 | 81.2 | 5.3 |
| 38 | 27.4 | 23.8 | 88.9 | 51.4 |
| 39 | 60.7 | 77.9 | 93.6 | 75.5 |
| 40 | 78.8 | 96.3 | 98.3 | 87.3 |
| 41 | 24.1 | 25.4 | 87.8 | 87.8 |
| 42 | 92.9 | 100 | 100 | 100 |
| 43 | 80 | 96 | 99.7 | 100 |
| 44 | 90.7 | 97.7 | 100 | 96.3 |
| 45 | 76.8 | 94.5 | 100 | 98.2 |
| 46 | 38 | 16.4 | 99.3 | 100 |
| 47 | 87.4 | 50.4 | 83.2 | 100 |
| 48 | 0.7 | 0 | 0 | 0 |
| 49 | 63.4 | 59.2 | 73.9 | 63.5 |
| 50 | 84.6 | 94.4 | 92.8 | 92.3 |
| 51 | 98.8 | 100 | 100 | 100 |
| 52 | 86 | 100 | 100 | 95 |
| 53 | 41 | 74.5 | 64.2 | 84.9 |
| 54 | 100 | 100 | 95.7 | 100 |
| 55 | 100 | 100 | 100 | 100 |
| 56 | 98.1 | 100 | 93 | 100 |
| 57 | 73.4 | 82.9 | 74.8 | 94.1 |
| 58 | 100 | 100 | 100 | 100 |
| 59 | 77.4 | 94.4 | 92.3 | 92.9 |
| 60 | 84 | 94.4 | 89.7 | 95.4 |
| 61 | 16.3 | 10.5 | 16.8 | 4.2 |
| 62 | 12.1 | 0 | 1.4 | 5.7 |
| 63 | 27.3 | 16.5 | 32.7 | 9.4 |
| 64 | 1 | 0 | 0 | 29.8 |
| 65 | 12 | 11.8 | 43.1 | 56.6 |
| 66 | 33.5 | 30 | 3.4 | 12.6 |
| 67 | 83.7 | 100 | 100 | 100 |
| 68 | 45.6 | 100 | 100 | 100 |
| 69 | 32.5 | 0 | 0 | 0 |
| 70 | 14.1 | 0.4 | 0 | 0 |
| 71 | 55.2 | 68.4 | 85.8 | 76.4 |
| 72 | 23.4 | 0 | 1.1 | 2.9 |
| 73 | 0.3 | 0 | 0 | 0 |
| 74 | 2 | 0 | 10.4 | 10.4 |
| 75 | 0 | 0 | 0 | 0 |
| 76 | 44.1 | 21.7 | 86.7 | 92.8 |
| 77 | 72.3 | 81.5 | 92.3 | 83.9 |
| 78 | 71.8 | 62.8 | 96.6 | 88.2 |
| 79 | 52 | 71 | 100 | 41.9 |
| 80 | 16.2 | 0.4 | 0.8 | 0 |
| 81 | 3.2 | 0 | 0 | 0 |
| 82 | 78.2 | 92.2 | 79.6 | 100 |
| 83 | 76.7 | 73.3 | 20.3 | 13.2 |
| 84 | 83.7 | 78.1 | 24.2 | 31.6 |
| 85 | 80.2 | 92.2 | 90.1 | 92.4 |
| 86 | 52 | 94.8 | 92.7 | 100 |
| 87 | 96.3 | 56.7 | 77 | 28.3 |
| 88 | 35.7 | 2.2 | 17.3 | 14.7 |
| 89 | 90.8 | 60.7 | 97.3 | 61.6 |
| 90 | 66.9 | 66.9 | 55.6 | 33.5 |
| 91 | 74.8 | 30.2 | 70 | 40.8 |
| 92 | 0 | 0 | 0 | 0 |
| 93 | 0 | 100 | 0 | 100 |
| 94 | 0 | 100 | 100 | 0 |
| 95 | 38.9 | 0 | 0 | 0.9 |

**Descriptive statistics**

**swine sector**

**Supplementary Figure S5: Distribution of exploitations and Full-Time Equivalent in pig sector**


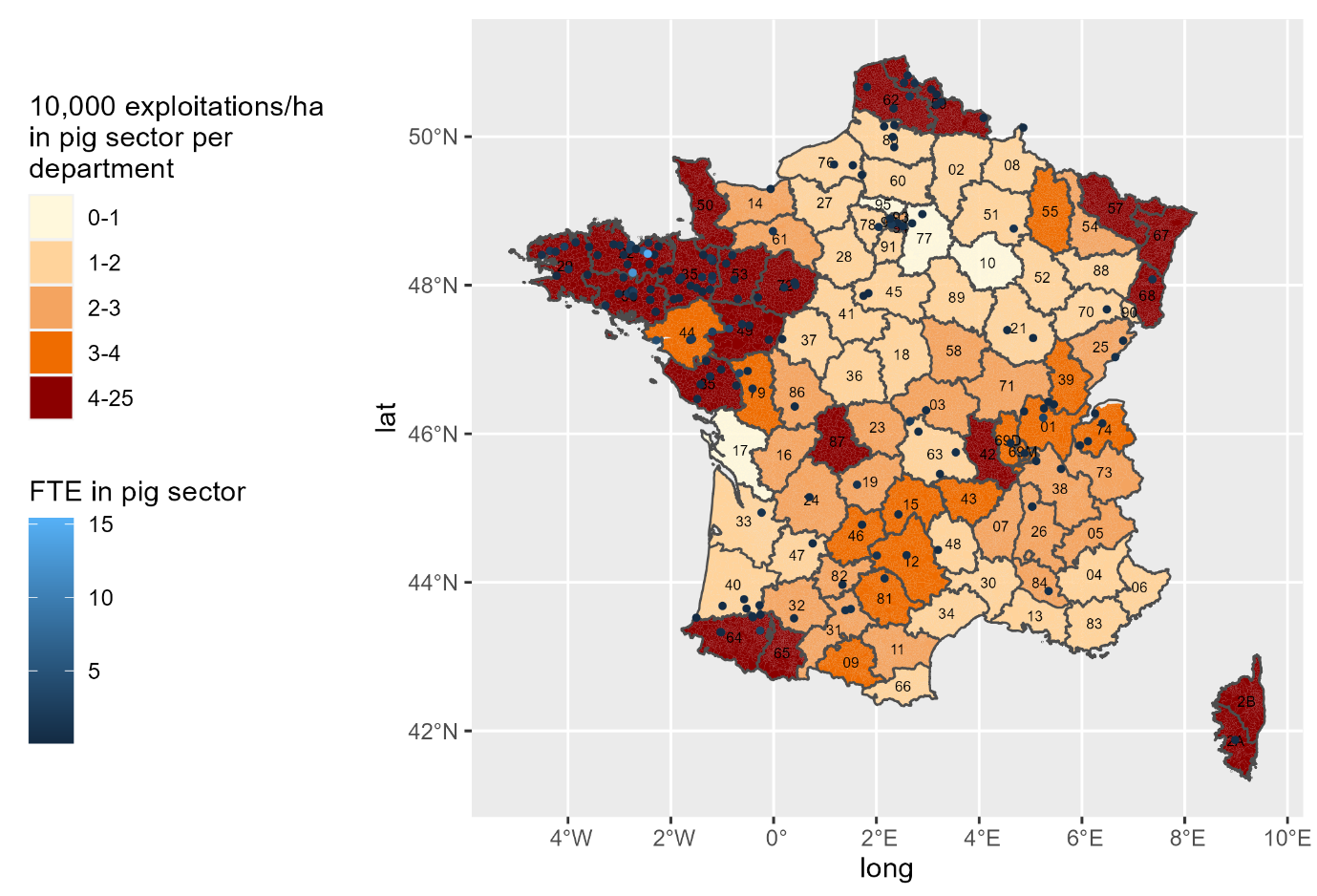


This figure was created by R software[14] version 4.0.3.

**Supplementary Table S6 : Percentage of underserved cantons by departement in swine sector according to travel time thresholds.**

| departement | underserved cantons 15 min threhsold (%) | underserved cantons 30 min threhsold (%) | Underserved cantons 45 min threhsold (%) | Underserved cantons 60 min threhsold (%) |
| --- | --- | --- | --- | --- |
| 1 | 73.1 | 66.1 | 100 | 23.8 |
| 2 | 100 | 100 | 100 | 100 |
| 3 | 96.1 | 89.5 | 88.1 | 68.7 |
| 4 | 100 | 100 | 100 | 100 |
| 5 | 100 | 100 | 100 | 100 |
| 6 | 100 | 100 | 100 | 100 |
| 7 | 100 | 100 | 100 | 100 |
| 8 | 100 | 100 | 100 | 90.3 |
| 9 | 100 | 100 | 100 | 100 |
| 10 | 100 | 100 | 100 | 100 |
| 11 | 100 | 100 | 100 | 100 |
| 12 | 80.8 | 83.9 | 94.2 | 100 |
| 13 | 100 | 100 | 100 | 100 |
| 14 | 95.4 | 86 | 90.6 | 100 |
| 15 | 100 | 100 | 100 | 100 |
| 16 | 100 | 100 | 100 | 100 |
| 17 | 100 | 100 | 100 | 100 |
| 18 | 100 | 100 | 100 | 100 |
| 19 | 81.2 | 79.8 | 100 | 100 |
| 21 | 96.2 | 100 | 100 | 100 |
| 22 | 67.5 | 44 | 38.9 | 11.6 |
| 23 | 100 | 92.7 | 92.7 | 70.1 |
| 24 | 94.8 | 94.8 | 91.6 | 88.5 |
| 25 | 100 | 100 | 79.1 | 79.1 |
| 26 | 96 | 93.4 | 100 | 100 |
| 27 | 100 | 100 | 100 | 100 |
| 28 | 100 | 100 | 100 | 93.3 |
| 29 | 79.5 | 86.8 | 81.6 | 100 |
| 2A | 100 | 100 | 100 | 100 |
| 2B | 100 | 100 | 100 | 100 |
| 30 | 100 | 100 | 100 | 100 |
| 31 | 94.9 | 81.7 | 63.6 | 100 |
| 32 | 100 | 96.9 | 96.9 | 100 |
| 33 | 91.9 | 79.6 | 78.5 | 100 |
| 34 | 100 | 100 | 100 | 100 |
| 35 | 69 | 63.2 | 55 | 63.5 |
| 36 | 100 | 100 | 100 | 100 |
| 37 | 100 | 100 | 100 | 100 |
| 38 | 97.9 | 88.9 | 100 | 100 |
| 39 | 100 | 100 | 100 | 92.8 |
| 40 | 92.6 | 93.5 | 93.5 | 100 |
| 41 | 100 | 100 | 100 | 100 |
| 42 | 100 | 100 | 100 | 100 |
| 43 | 100 | 100 | 100 | 100 |
| 44 | 84 | 37.7 | 9.7 | 7 |
| 45 | 100 | 100 | 100 | 100 |
| 46 | 92.6 | 100 | 100 | 100 |
| 47 | 88.5 | 100 | 100 | 100 |
| 48 | 100 | 100 | 100 | 100 |
| 49 | 72.8 | 66.4 | 33.3 | 28.6 |
| 50 | 100 | 100 | 100 | 90.8 |
| 51 | 81.3 | 81.3 | 81.3 | 100 |
| 52 | 100 | 100 | 97.5 | 100 |
| 53 | 92.6 | 85.8 | 81.2 | 73.6 |
| 54 | 100 | 100 | 100 | 100 |
| 55 | 100 | 100 | 100 | 100 |
| 56 | 68.9 | 63.6 | 54.1 | 38.6 |
| 57 | 100 | 100 | 100 | 100 |
| 58 | 100 | 100 | 100 | 100 |
| 59 | 82.1 | 74 | 78.2 | 97.3 |
| 60 | 100 | 100 | 100 | 89.6 |
| 61 | 89.2 | 69 | 61.8 | 100 |
| 62 | 92 | 88.3 | 87 | 93.9 |
| 63 | 61.9 | 70.3 | 70.3 | 81.7 |
| 64 | 88.5 | 75.6 | 79.4 | 100 |
| 65 | 100 | 100 | 100 | 100 |
| 66 | 100 | 100 | 100 | 100 |
| 67 | 100 | 100 | 100 | 100 |
| 68 | 85.1 | 50.2 | 99.7 | 100 |
| 69 | 91.1 | 72.7 | 100 | 95.1 |
| 70 | 100 | 88.5 | 100 | 100 |
| 71 | 100 | 100 | 100 | 87 |
| 72 | 76.3 | 62.9 | 75.3 | 94.7 |
| 73 | 100 | 100 | 100 | 100 |
| 74 | 81.4 | 87.5 | 100 | 100 |
| 75 | 0 | 0 | 0 | 0 |
| 76 | 88.9 | 90.2 | 100 | 100 |
| 77 | 82.9 | 82.9 | 83.3 | 49.8 |
| 78 | 91.6 | 33.3 | 0 | 0 |
| 79 | 77.2 | 74.1 | 74.1 | 61.6 |
| 80 | 92.4 | 96.2 | 100 | 100 |
| 81 | 99.3 | 99.3 | 95.5 | 95.5 |
| 82 | 91.5 | 100 | 88.3 | 100 |
| 83 | 100 | 100 | 100 | 100 |
| 84 | 77.6 | 67.9 | 61.8 | 100 |
| 85 | 88.1 | 72.5 | 83.1 | 57.7 |
| 86 | 100 | 100 | 100 | 100 |
| 87 | 100 | 100 | 100 | 100 |
| 88 | 100 | 100 | 100 | 100 |
| 89 | 100 | 100 | 100 | 100 |
| 90 | 100 | 100 | 100 | 100 |
| 91 | 100 | 100 | 71.5 | 0 |
| 92 | 0 | 0 | 0 | 0 |
| 93 | 100 | 0 | 0 | 0 |
| 94 | 79.6 | 0 | 0 | 0 |
| 95 | 100 | 100 | 57.6 | 0 |

**Descriptive statistics**

**poultry sector**

**Supplementary Figure S7: Distribution of exploitations and Full-Time Equivalent in poultry sector**


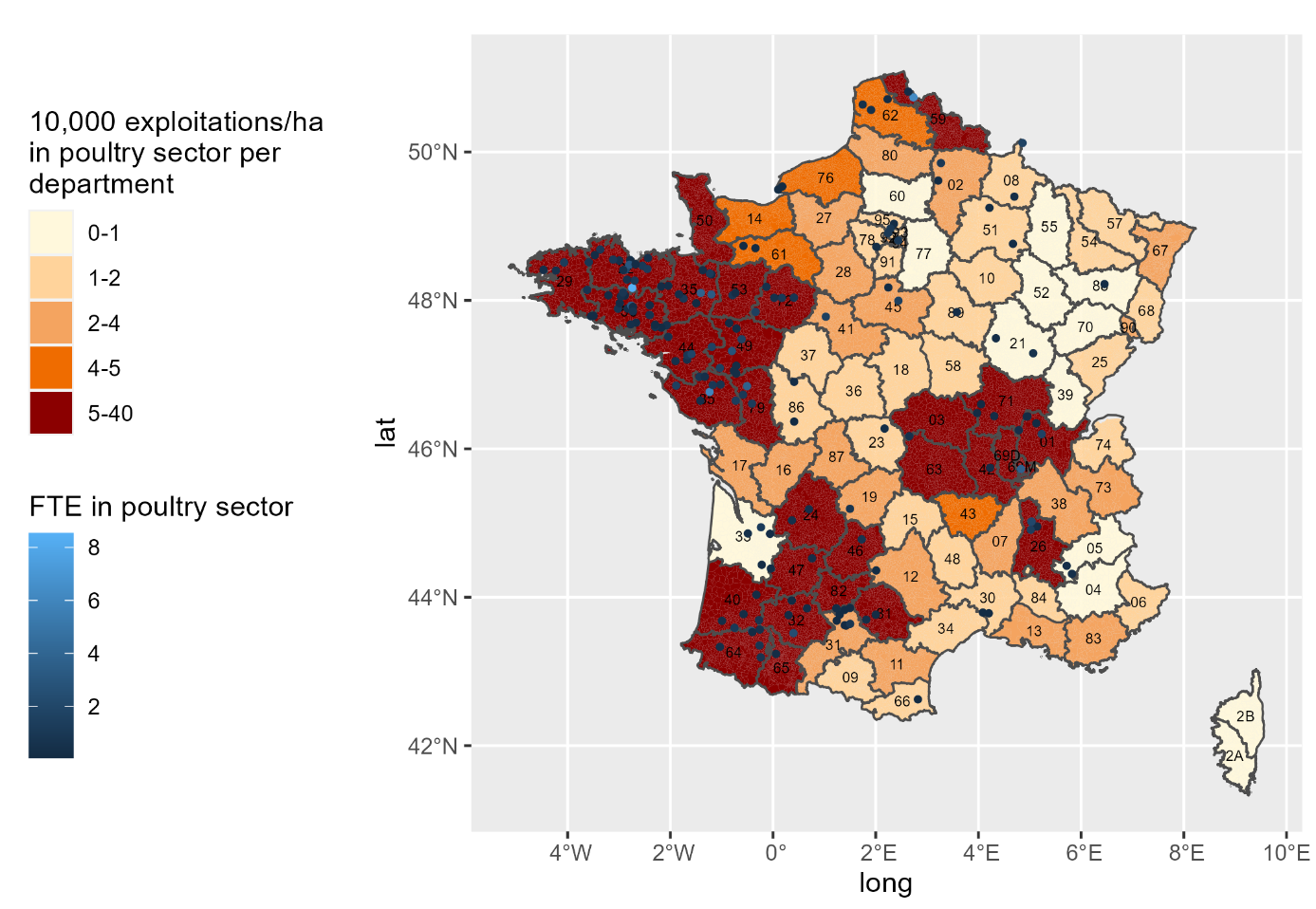


This figure was created by R software[14] version 4.0.3.

**Supplementary Table S8 : Percentage of underserved cantons by departement in poultry sector according to travel time thresholds.**

| departement | underserved cantons 15 min threhsold (%) | underserved cantons 30 min threhsold (%) | Underserved cantons 45 min threhsold (%) | Underserved cantons 60 min threhsold (%) |
| --- | --- | --- | --- | --- |
| 1 | 83.9 | 99.2 | 100 | 100 |
| 2 | 95.3 | 95.1 | 95.5 | 100 |
| 3 | 96.1 | 91.7 | 100 | 100 |
| 4 | 100 | 74.9 | 96.7 | 100 |
| 5 | 73.5 | 81.1 | 100 | 100 |
| 6 | 100 | 89.5 | 100 | 100 |
| 7 | 100 | 78.5 | 84.3 | 98.9 |
| 8 | 100 | 80 | 100 | 100 |
| 9 | 100 | 77.9 | 95.2 | 100 |
| 10 | 100 | 100 | 100 | 100 |
| 11 | 100 | 100 | 100 | 100 |
| 12 | 100 | 96.6 | 100 | 100 |
| 13 | 100 | 94.3 | 94.3 | 98.2 |
| 14 | 100 | 98.6 | 100 | 100 |
| 15 | 100 | 93.4 | 93.4 | 93.4 |
| 16 | 100 | 98.1 | 100 | 100 |
| 17 | 100 | 89.3 | 94.7 | 95.3 |
| 18 | 100 | 95 | 100 | 100 |
| 19 | 93.4 | 71.6 | 100 | 100 |
| 21 | 82.4 | 100 | 96.7 | 100 |
| 22 | 48.7 | 96.6 | 100 | 100 |
| 23 | 100 | 100 | 100 | 100 |
| 24 | 95.5 | 95 | 99.4 | 100 |
| 25 | 100 | 88.3 | 100 | 100 |
| 26 | 81.7 | 99.5 | 99.5 | 99.1 |
| 27 | 100 | 93.9 | 96.3 | 96.3 |
| 28 | 100 | 100 | 100 | 100 |
| 29 | 85.1 | 94.6 | 96.1 | 100 |
| 2A | 100 | 100 | 100 | 100 |
| 2B | 100 | 100 | 100 | 100 |
| 30 | 94.6 | 76.7 | 95.1 | 100 |
| 31 | 95.2 | 71.9 | 100 | 100 |
| 32 | 92.6 | 100 | 100 | 91.8 |
| 33 | 76.6 | 90.4 | 71.4 | 76.8 |
| 34 | 100 | 90.1 | 100 | 100 |
| 35 | 57.9 | 93.3 | 100 | 100 |
| 36 | 100 | 93.9 | 100 | 100 |
| 37 | 100 | 91.5 | 91.5 | 100 |
| 38 | 100 | 94.8 | 100 | 100 |
| 39 | 100 | 74.2 | 100 | 100 |
| 40 | 100 | 100 | 100 | 100 |
| 41 | 87.1 | 100 | 100 | 100 |
| 42 | 100 | 89.9 | 99.3 | 99.5 |
| 43 | 100 | 80.2 | 100 | 90.3 |
| 44 | 62.7 | 88.7 | 94.1 | 100 |
| 45 | 78.4 | 99.3 | 99.3 | 100 |
| 46 | 92.6 | 90.2 | 100 | 100 |
| 47 | 100 | 88.8 | 100 | 100 |
| 48 | 100 | 99.2 | 100 | 99.2 |
| 49 | 80.2 | 97.8 | 100 | 100 |
| 50 | 100 | 98.7 | 100 | 100 |
| 51 | 78.9 | 61.9 | 100 | 79.3 |
| 52 | 100 | 82.7 | 100 | 100 |
| 53 | 91.3 | 98.7 | 100 | 100 |
| 54 | 100 | 100 | 100 | 100 |
| 55 | 100 | 88.1 | 75.2 | 88.1 |
| 56 | 80.8 | 100 | 100 | 100 |
| 57 | 100 | 100 | 100 | 100 |
| 58 | 100 | 88.7 | 100 | 100 |
| 59 | 88.8 | 93.8 | 91.7 | 100 |
| 60 | 100 | 85.1 | 85.1 | 100 |
| 61 | 100 | 78.5 | 97.5 | 97.5 |
| 62 | 82.1 | 95.7 | 95.7 | 95.7 |
| 63 | 100 | 100 | 100 | 100 |
| 64 | 100 | 99.2 | 100 | 100 |
| 65 | 100 | 100 | 100 | 100 |
| 66 | 100 | 100 | 100 | 96.9 |
| 67 | 100 | 100 | 100 | 100 |
| 68 | 99.7 | 81.5 | 81.8 | 100 |
| 69 | 81.5 | 89.9 | 100 | 100 |
| 70 | 100 | 75.9 | 87.9 | 87.9 |
| 71 | 100 | 99.2 | 100 | 100 |
| 72 | 88.6 | 90.4 | 100 | 100 |
| 73 | 100 | 100 | 100 | 100 |
| 74 | 100 | 100 | 100 | 100 |
| 75 | 0 | 0 | 0 | 0 |
| 76 | 100 | 98.5 | 100 | 100 |
| 77 | 100 | 100 | 98.6 | 100 |
| 78 | 91.4 | 57.9 | 100 | 100 |
| 79 | 87.1 | 100 | 100 | 90.8 |
| 80 | 100 | 100 | 100 | 100 |
| 81 | 100 | 95 | 95 | 100 |
| 82 | 93.5 | 100 | 100 | 100 |
| 83 | 100 | 88.2 | 88.2 | 98.4 |
| 84 | 100 | 100 | 100 | 100 |
| 85 | 82.1 | 100 | 100 | 98.1 |
| 86 | 94.2 | 96.4 | 96.4 | 100 |
| 87 | 100 | 100 | 91.5 | 100 |
| 88 | 100 | 87.9 | 78.5 | 100 |
| 89 | 100 | 94.9 | 94.9 | 94.9 |
| 90 | 100 | 57.7 | 57.7 | 84.4 |
| 91 | 100 | 100 | 100 | 100 |
| 92 | 100 | 100 | 100 | 100 |
| 93 | 0 | 0 | 0 | 0 |
| 94 | 75.2 | 100 | 100 | 100 |
| 95 | 98.5 | 100 | 100 | 78.7 |
